# Vehicle Detection in Urban Traffic Surveillance Images Based on Convolutional Neural Networks with Feature Concatenation

**DOI:** 10.3390/s19030594

**Published:** 2019-01-30

**Authors:** Fukai Zhang, Ce Li, Feng Yang

**Affiliations:** School of Mechanical Electronic and Information Engineering, China University of Mining and Technology, Beijing, Beijing 100083, China; celi@cumtb.edu.cn (C.L.); yangf@cumtb.edu.cn (F.Y.)

**Keywords:** vehicle detection, feature concatenation, default box, convolutional neural network, real-time

## Abstract

Vehicle detection with category inference on video sequence data is an important but challenging task for urban traffic surveillance. The difficulty of this task lies in the fact that it requires accurate localization of relatively small vehicles in complex scenes and expects real-time detection. In this paper, we present a vehicle detection framework that improves the performance of the conventional Single Shot MultiBox Detector (SSD), which effectively detects different types of vehicles in real-time. Our approach, which proposes the use of different feature extractors for localization and classification tasks in a single network, and to enhance these two feature extractors through deconvolution (D) and pooling (P) between layers in the feature pyramid, is denoted as DP-SSD. In addition, we extend the scope of the default box by adjusting its scale so that smaller default boxes can be exploited to guide DP-SSD training. Experimental results on the UA-DETRAC and KITTI datasets demonstrate that DP-SSD can achieve efficient vehicle detection for real-world traffic surveillance data in real-time. For the UA-DETRAC test set trained with UA-DETRAC trainval set, DP-SSD with the input size of 300 × 300 achieves 75.43% mAP (mean average precision) at the speed of 50.47 FPS (frames per second), and the framework with a 512 × 512 sized input reaches 77.94% mAP at 25.12 FPS using an NVIDIA GeForce GTX 1080Ti GPU. The DP-SSD shows comparable accuracy, which is better than those of the compared state-of-the-art models, except for YOLOv3.

## 1. Introduction

Automatic analysis of vehicle activities in urban traffic surveillance is an important and urgent issue due to a large number of vehicle traffic rule violations and their adverse effects on daily traffic management. Compared with traditional machine learning tasks, deep learning-based methods have made great breakthroughs in traffic surveillance techniques, and have achieved good performance in practical applications such as vehicle detection, feature extraction and vehicle track identification [1,2,3,4,5]. In these areas of research, accurate and real-time vehicle detection and preliminary classification are the most fundamental and important work.

**Figure 1:**
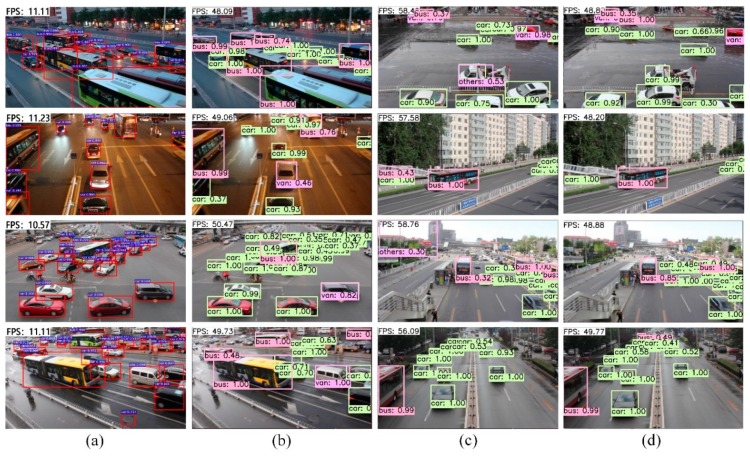
Conventional methods vs. the proposed DP-SSD300 framework on UA-DETRAC dataset. (**a**) Faster R-CNN with low FPS for vehicle detection; (**b**) DP-SSD300 with high FPS for vehicle detection; (**c**) In SSD300, one white vehicle is labeled with two bounding boxes, and some small objects are error detected or miss detected; (**d**) In DP-SSD300, the white vehicle is well labeled, and small objects are well detected.

Among the various vehicle detection algorithms of traffic surveillance, convolutional neural network (CNN) based methods have been widely applied and can be categorized into region-based and regression-based approaches. For region-based CNNs, Regions with CNN features (R-CNN) [6], Spatial Pyramid Pooling Network (SSP-Net) [7], Fast R-CNN [8] and Faster R-CNN [9] are some recent advances often utilized in vehicle detection. These approaches, although achieving state-of-the-art accuracy through the improvement from Selective Search [10] to Region Proposal Network (RPN) [9], are too computationally intensive for bounding boxes to be too slow for real-time or near real-time. As shown in Figure 1a, which demonstrates the detection results of the fastest high-accuracy region-based method (Faster R-CNN), the detection frame rate of each image does not exceed 12 FPS, which is not enough for real-time detection in traffic surveillance. To achieve real-time detection performance, a wide range of detectors have been put forward by attacking either classifiers or region proposals. However, most of these attempts increase the speed at the expense of the detection accuracy.

With the advance of novel methods for predicting bounding boxes and class probabilities directly in a single neural network, a regression-based You Only Look Once (YOLO) approach [11] processes images in real-time at 45 FPS, but with less accuracy than Faster R-CNN. To further increase the speed vs accuracy trade-off, SSD [12] adopts an anchor mechanism similar to Faster R-CNN, and produces predictions through several feature maps of different aspect ratios and scales. Although it performs well in both speed and accuracy, SSD neglects the relationship between different layers of the feature pyramid. Thus, SSD has a relatively poor performance in traffic surveillance of small vehicles, and produces multiple scale bounding boxes for one vehicle. Specifically, in Figure 1c, two boxes are produced on one car, and the bus in the distance is not detected.

**Figure 2:**
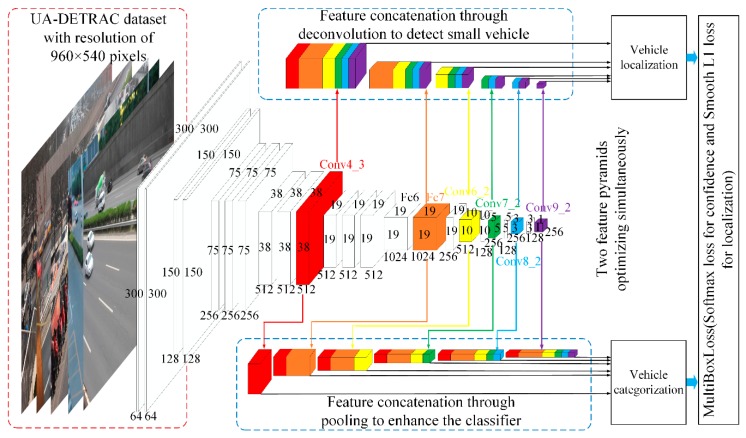
Training architecture of DP-SSD for vehicle detection. Our DP-SSD model utilizes conventional SSD, but divides the feature pyramid for detection into two parts: feature pyramid for localization and feature pyramid for classification. For localization, we enhance the representation power of lower layers by concatenating feature maps through deconvolution. The classification feature pyramid concatenates feature maps of the upper layers through pooling.

Inspired by the conclusions of previous research, in this paper, we propose an enhanced framework DP-SSD based on a single deep neural network, as shown in Figure 2, for accurate and real-time vehicle detection in urban traffic surveillance. Specifically, DP-SSD is based on conventional SSD, and expands the feature pyramid for vehicle detection into two parts: feature pyramid for localization and feature pyramid for classification. The localization feature pyramid concatenates the upper layer feature maps into lower layer features through deconvolution, which is able to predict all the bounding boxes of vehicles. In the process of predicting, a set of default boxes over multiple scales and aspect ratios are applied to better match the vehicle shape. The latter classification feature pyramid concatenated feature maps of the upper layers through pooling can precisely infer vehicle categories for each positive default box. Therefore, DP-SSD is derived from the idea of feature concatenation through deconvolution (D) and pooling (P), respectively. Compared with the complex rainbow concatenation in R-SSD [13], the novelty of DP-SSD is that two different concatenated feature pyramids are used to perform localization task and classification task respectively, which improves the detection effect while reducing the computational complexity. Section 4 verifies the excellence of DP-SSD. This is because previous works [14] have shown that the upper feature maps are able to achieve high recall but have poor localization in predictions, and the lower feature maps are capable of improving the localization accuracy but with reduced recall. It is noteworthy that both feature pyramids take advantage of the relationship between different layers and increase the number of channels for each feature map layer. In this way, the proposed method can prevent various boxes for one vehicle and predict smaller vehicles in real-time.

The main contributions of our work are summarized as follows:

(1) We propose for the first time the use of different feature extractors (enhanced through deconvolution and enhanced through pooling) for the localization and classification tasks, respectively, in the vehicle detection process, which results in a significant improvement of speed and detection accuracy trade-off.
(2) In order to enhance the representation power of feature pyramids, we concatenate lower feature maps of basic feature pyramid through deconvolution to precisely produce vehicle bounding boxes, and concatenate upper feature maps of basic feature pyramid through pooling to accurately and simultaneously classify vehicle categories.
(3) For each concatenated feature pyramid, we increase the number of channels for each feature layer in the pyramid. In this way, the multiple scale boxes for one vehicle can be effectively eliminated.
(4) As for the size of default box, we allow the predictions to produce smaller bounding boxes than conventional SSD by adjusting the min_size and max_size parameters, thus can accurately detect small vehicles in real-time.

This paper is organized as follows: in Section 2, we briefly discuss the related works of vehicle detection in the area of deep learning, and then propose a feature concatenation framework, which we call DP-SSD, in Section 3. In Section 4, the experiments and results of the proposed method are presented. Finally, this paper is concluded in Section 5.

## 2. Related Work

With the continuous breakthroughs in deep networks on image classification tasks, such as those mentioned in References [15,16,17,18], a wide variety of object detection algorithms using CNNs and their applications to vehicle detection have achieved dramatic progress. Here, we briefly introduce the latest research on deep CNNs for object detection and vehicle detection, respectively.

### 2.1. Deep CNNs for Object Detection

There are two established classes of algorithms for object detection in the field of deep CNNs, one based on the region proposal method and the other based on the idea of regression. Region proposal-based methods first produce candidate regions in an image and then classify each of them, are the beginning of object detection in deep CNNs. The original R-CNN [6] applies high-capacity CNNs to bottom-up candidate regions proposed through a Selective Search algorithm [10], which effectively improves accuracy. Although R-CNN combines region proposals with CNNs, it leads to heavy computational costs without sharing convolutional layers. SPP-Net [7] equips the deep network with a spatial pyramid pooling strategy, which is more robust to region size or scale, to speed up the original R-CNN significantly. Fast R-CNN [8] improves the disadvantages of R-CNN and SPP-Net so that it can update all network layers end-to-end by using a multi-task loss for both localization and classification, which was advocated in References [19,20]. Faster R-CNN [9] first replaces Selective Search algorithm by a Region Proposal Network (RPN), and merges the RPN with Fast R-CNN into a single network by sharing convolutional layers using “attention” mechanisms, which achieves towards real-time detection performance with guaranteed accuracy. Among these methods, the top-most layer is used to detect objects at multi scales. Although powerful, a single layer is difficult to consider all shapes and scales of the objects. Inside-Outside Net (ION) [21] concatenates feature maps from several layers by L2-normalization [22] to produce fixed-length feature map for object proposals, which enhances the multi-scale representation power. In Reference [23], HyperNet (Hierarchical Network, HyperNet) compresses hierarchical feature maps into a uniform space, which is shared both in generating proposals and detecting objects. In all these methods, although detection accuracy has been greatly improved, speed is still a problem, and real-time detection is impossible.

Regression based methods, which significantly improve the speed of object detection using a single neural network, have attracted much attention. YOLO [11], by dividing an input image into multiple grids and predicts bounding boxes and confidence directly in each grid, is extremely fast. While YOLO makes numerous localization errors compared to region proposal-based algorithms, an improved model, which is called YOLOv2, removes the fully connected layers and uses an anchor box to offer a trade-off between speed and accuracy [24]. SSD in Reference [12], which builds a set of default boxes of different aspect ratios and scales, combines multiple convolution layers with different resolutions for object detection, and performs well in both speed and accuracy. To supplement the insufficient performance of SSD on small object, Deconvolutional Single Shot Detector (DSSD) [25] applies a deconvolution module to enhance additional contexts, where VGGNet [17] is replaced by Residual Network (ResNet) [18], which increases the accuracy at the expense of speed. In [13], an enhanced SSD method by concatenating feature maps in both lower layers and upper layers is proposed, which improves the accuracy of SSD and is denoted as R-SSD. A novel single-shot based object detection method, called RefineDet, is presented in Reference [26], which simultaneously optimizes an anchor refinement module and an object detection module to efficiently detect objects. In Reference [27], Structure Inference Network (SIN), which considers the context of scene contextual information and object relationships in a single image, is proposed to take object detection as a problem of graph structure inference, and obtains a desirable output. To solve the scale problem in object detection, [28] presents a scale-transferrable detection network for multi-scale object detection, which based on a dense convolutional network. Experiments on the PASCAL VOC 2007 and MS COCO datasets show that the proposed method achieves obvious improvements compared to existing methods. An enhanced version of YOLOv2, which is denoted as YOLOv3 in Reference [29], uses a residual network and combines multi-scale prediction network to improve both speed and accuracy for object detection. Most of these methods achieve real-time detection, and the accuracy is progressively improved.

### 2.2. Deep CNNs for Vehicle Detection

Conventional vehicle detection algorithms are intrinsically related to background subtraction problems [30], which have made great breakthroughs in recent years. A Principal Component Pursuit (PCP) method is proposed to detect moving objects in Reference [31], which recovers the low-rank and sparse matrices via convex optimization, and successfully removes shadows from detected objects. Reference [32] uses a Stable Outlier Pursuit (SOP) algorithm to detect moving objects, which achieves robust decomposition while the outliers corrupt entire columns. In Reference [33], a vehicle detection approach based on probabilistic neural networks is presented, which can accurately detect moving vehicles in both high bit-rate and low bit-rate video streams. However, the application of deep CNNs in vehicle detection mainly depends on state-of-the-art object detection algorithms, and is improved based on the practical application scenarios and data characteristics. A novel framework named Evolving Boxes [34], which obtains significant boost on mAP compared to Faster R-CNN and achieves 9–13 FPS speed, is proposed. Evolving Boxes consists of three networks called Deep Convolutional Network (DCN), Proposal Network (PN), and Fine-Tuning Network (FTN), and evaluates on DETRAC benchmark. In Reference [2], a scale-insensitive convolutional neural network (SINet) is adopted for vehicle detection with various scales on KITTI benchmark, which prominently improves the detection accuracy of extremely small objects with a speed up to 37 FPS. It is noteworthy, SINet can be applied to any deep network and can be trained end-to-end. A multi-task vehicle detection framework with region-of-interest voting scheme is presented in Reference [1], which experiments on KITTI benchmark and PASCAL2007 vehicle dataset, respectively, and achieves extraordinary performance compared with other excellent frameworks in vehicle detection. In Reference [35], a scene-adaptive algorithm by employing deep convolutional neural network is introduced for vehicle detection, which can be applied in various scenes and demonstrates a higher detection rate than existing machine-learning-based method. In Reference [36], an approach by combining a bioinspired image enhancement method with a weighted feature fusion technique is introduced for nighttime vehicle detection, which can be applied in various scenes and demonstrates a higher detection rate than some state-of-the-art algorithms. A Multi-Perspective Tracking (MPT) framework in Reference [37], which is conducted on a MPT dataset, learns the surroundings of the vehicle using an iterative search procedure for detection, and a Siamese convolutional neural network is employed for feature extraction. Experiments show that MPT achieves good performance of vehicle detection from multiple perspective. For higher detection performance, recently, a unified framework called Detection and Annotation for Vehicles (DAVE) [38] employs a two-stage architecture of a shallow fully convolutional network and an extended GoogLeNet [16] for vehicle detection and attributes learning in urban traffic surveillance. DAVE, which was trained on the CompCars dataset [39] and used to evaluate the self-collected UTS dataset, PASCAL VOC 2007 car dataset [40] and LISA 2010 dataset [4], respectively, obtains significant improvements over other published methods.

In addition to road vehicle detection, vehicle detection utilizing deep CNNs in aerial images has also been rapidly developed. In Reference [41], an approach named Oriented_SSD is proposed to generate arbitrary-oriented bounding boxes for vehicle detection in aerial images. It is based on a single convolutional network SSD [12] and evaluated on the DLR Vehicle Aerial dataset and VEDAI dataset, respectively. Oriented_SSD, which is more accurate and robust than SSD, can detect vehicle location and orientation simultaneously. To ensure good performance on small vehicles with complex backgrounds in aerial vehicle detection, an improvement algorithm based on Faster R-CNN is presented in Reference [42], which is applied to the Munich vehicle dataset using a hyper region proposal network (HPRN) and achieves great improvements in accuracy compared to the existing methods. In Reference [43], a coupled R-CNN method, which combines an accurate-vehicle-proposal-network and a vehicle attribute learning network, is proposed to fast and accurate detect vehicles. A vehicle detection algorithm using spatial pyramid pooling-based convolutional neural networks in proposed [44], which can better adapt to input images of different sizes to learn of the multi-scale characteristics of objects.

This paper is inspired by the conclusion that the lower-layer feature map has higher location precision, and the upper-layer feature map has better classification accuracy. Therefore, we take the advantages of SSD [12] and the idea of feature concatenation to propose our method and achieve good results in vehicle detection.

## 3. The Proposed Vehicle Detection Method

The framework of our proposed vehicle detection method is a single deep neural network, which directly performs to regress the vehicle locations and classify vehicle categories simultaneously. As illustrated in Figure 2, the network layers of the detection approach are based on an extended architecture (VGG-16) with converting the fully connected layers fc6 and fc7 to convolutional layers and adding auxiliary convolutional feature layers (e.g., conv6_1, conv6_2, conv7_1, conv7_2, conv8_1, conv8_2, conv9_1, and conv9_2) to the end of the fc7 layer to produce more lower resolution feature maps, which we call the base network. At prediction time, conventional methods use conv4_3, fc7, conv6_2, conv7_2, conv8_2 and conv9_2 as the basic feature pyramid to predict both location and confidences. To make the detection more accurate, we increase the number of feature maps in each feature pyramid layers by concatenating feature maps of lower layers through deconvolution to enhance the localization performs and concatenating feature maps of the upper layers through pooling to strengthen the categorization performs. This method proves to be able to enhance the performance of vehicle location and the presence of each vehicle categories, respectively. The details of feature concatenation and the enhanced default box for predictions will be described in the following subsections.

### 3.1. Feature Concatenation

Searching the whole image to locate potential vehicle positions and classify its categories in a single feature map is limited by the detection accuracy. The conventional SSD method combines both lower and upper feature maps in the classifier network to address this problem, but the relationship between each feature map is not considered, which lowers the efficiency to some extent. Particularly for the specific application, we expect significantly increased detection accuracy can be achieved in the case of real-time. Our proposed feature concatenation method is a way to enrich the representation power of the feature maps in different layers (the original conv4_3, fc7, conv6_2, conv7_2, conv8_2 and conv9_2 layers), which aims to not only precisely localize all the vehicle-like objects but also accurately classify the presence of each vehicle-like objects in real-time.

#### 3.1.1. Feature Concatenation through Deconvolution for Localization

We concatenate feature maps of the lower layers through deconvolution, as depicted in the top part of Table 1, to enhance representation power of lower feature maps. From up to low, the first convolutional layer (conv9_2) remains unchanged as the first new deconvolution layer. In the second stage, we filter the new deconvolution layer (conv9_2) with 256 kernels of size 3 × 3 and a stride of one pixel to the same size with the second convolutional layer (conv8_2), which we call deconvolution feature map (deconv9_2). Then, after a batch normalization step with both layers [45], we concatenate convolutional layer (conv8_2) with deconvolution feature map (deconv9_2), to enrich the representation power of lower feature map (conv8_2) by increasing the number of feature maps efficiently. In the third stage, the third convolutional layer (conv7_2) takes as input the concatenated feature maps obtained from the previous layer and filters them with 256 kernels of size 3 × 3 and a stride of one pixel to generate deconvolution feature map (deconv8_2), afterwards, we concatenate both conv7_2 and deconv8_2 in the same way as the second stage. Meanwhile, the feature maps at the third stage consists of three feature maps. Similarly, the fourth (conv6_2), fifth (fc7), and sixth (conv4_3) convolutional layers are concatenated with deconvolution feature map (deconv7_2) filtered by 256 kernels of size 2 × 2 and a stride of two pixels, deconvolution feature map (deconv6_2) filtered by 512 kernels of size 1 × 1 and a stride of two pixels, and deconvolution feature map (defc7) filtered by 1024 kernels of size 2 × 2 and a stride of two pixels, respectively. It is noteworthy that a normalization step needs to be performed before concatenating feature maps. This is because the size of the feature map in different layers are quite different.

In addition, the number of channels in the feature pyramid is increased efficiently. By concatenating feature maps, from up to low, each of deconvolution layer contains 256, 512, 768, 1280, 2304 and 2816 feature maps, respectively. This method will directly achieve precise localization, especially for small vehicles from whole image or frames in video.

#### 3.1.2. Feature Concatenation through Pooling for Categorization

The bottom part of Table 1 shows the architecture details of pooling concatenation. In order to make the network be trained with more meticulous vehicle features, from low to up, we use the first convolutional layer (conv4_3) unchanged as the first new pooling layer. Next, we pool the new convolutional layer (conv4_3) with kernel of size 2 × 2 and a stride of two pixels by means of max pooling, which we call pooling feature map (pooling4_3). At the same time, we need to ensure the same size as the second convolutional layer (fc7). Then, by utilizing a batch normalization step, we concatenate convolutional layer (fc7) with pooling feature map (pooling4_3) to strengthen the representation power of upper feature map (fc7) by increasing the number of feature maps efficiently. Similar to the deconvolution process, in the next, the third (conv6_2), fourth (conv7_2) and fifth (conv8_2) convolutional layers are concatenated with pooling feature maps poolingfc7, pooling6_2 and pooling7_2 that pooled by kernel of size 2 × 2 and a stride of two pixels by means of max pooling respectively. At last, we concatenate the sixth (conv9_2) convolutional layers with pooling feature map (pooling8_2) pooled by kernel of size 3 × 3 and a stride of one pixel by means of max pooling. In this way, the network with large receptive fields can have enhanced representation power for vehicle classification. Therefore, from low to up, each of pooling layer contains 512, 1536, 2048, 2304, 2560 and 2816 feature maps, respectively.

In all of the above, we use a single deep neural network for detecting vehicles, but different feature extractors are used for the localization task and classification task, respectively. The classifier used in the localization task is obtained by concatenating feature maps of lower layers through deconvolution, and the classifier for the classification task combines the feature maps of upper layers through pooling. The advantage of this method is that the network can executed with information from other layers in each of the feature maps, which will precisely detect vehicles in real-time.

### 3.2. Default Box with Feature Concatenation

**Figure 3:**
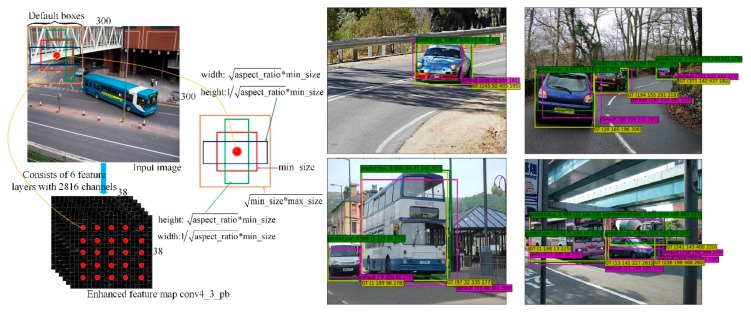
**Left**: Feature map concatenation and the calculation of default boxes with multiple aspect ratios and scales in the process; **Right**: Example detections using DP-SSD300 with new default box size in Table 2 on PASCAL VOC 2007 car dataset.

For vehicle detection predictions, we adopt a small convolution kernel of 3 × 3 to filter the feature map output by the top of the network to produce a score for the vehicle category and a rectangular offset relative to the default box coordinates. Motivated by References [22,46,47], the conventional SSD method uses a feature pyramid that includes both the lower and upper feature maps to fit the default box. Previous work [13] has demonstrated that the greater the number of channels in the feature map, the better the performance becomes. Simultaneously, Reference [14] showed that the upper feature maps can find the object of interest with high recall, and the lower feature maps can better localize the object of interest. Inspired by these works, we use concatenated feature maps of different resolutions obtained by deconvolution for predicting vehicle localization, and feature maps of multiple layers concatenated through pooling for calculating confidences, respectively. Figure 3 left shows the concatenated feature map conv4_3_pb consisting of six feature layers including 2816 channels, which is used in our framework, and other feature maps are similar.

Our approach, concatenation-based feature pyramid, fits the predicted bounding boxes into a set of default boxes through different scales and aspect ratios at each feature map location. To generate default boxes that can match the specific vehicle shape, suppose m feature maps of different resolutions are used, and two parameters of the default box scale min_size and max_size are defined as follows:

(1) $$\min\_\mathrm{size}=\frac{\min\_\dim\times \mathrm{ratio}}{100}$$

(2) $$\max\_\mathrm{size}=\frac{\min\_\dim\times \left(\mathrm{ratio}+\mathrm{step}\right)}{100}$$

(3) $$\mathrm{where}\;\mathrm{step}=\frac{\max\_\mathrm{ratio}-\min\_\mathrm{ratio}}{m-2}$$

Here, min_dim is the size of input image, meanwhile we set min_raito as 15 and max_ratio as 90. Starting at 15, we generate a set of data regularly spaced at step intervals between 15 and 90, and we assign them to ratio respectively. Table 2 and Table 3 show the specific values of min_size and max_size in several feature layers of different resolutions for the 300 and 512 input models, respectively. In addition, we use 6 aspect ratios of 1:1, 1:1, 1:2, 2:1, 1:3, 3:1 for the default boxes in each feature map location. For the two 1:1 aspect ratios, we compute the scales as min_size and $\sqrt{\min\_\mathrm{size}\times \max\_\mathrm{size}}$, respectively. Besides, for the rest aspect ratios, we define the width as $\sqrt{\mathrm{aspect}\_\mathrm{ratio}}\times \min\_\mathrm{size}$, and height as $\min\_\mathrm{size}/\sqrt{\mathrm{aspect}\_\mathrm{ratio}}$ for each default box. In detail, Figure 3 left also shows the calculation of default boxes with different aspect ratios and scales.

### 3.3. Deep Nets Training

The vehicle detection network with feature concatenation can be trained end-to-end by stochastic gradient descent (SGD) [48]. To make a full training, we randomly initialize all layers used for predicting confidences by drawing weights from the Xavier algorithm [49]. All other layers (i.e., predicting localization layers) are initialized by the pretrained truncated VGG model for ImageNet classification [9]. The reason is that offsets are independent of category, and the classification layers cannot share weights due to different number of categories.

At training time, we assign a positive label to default boxes by matching them to the ground truth in two ways: (i) each of the ground truth box with the best jaccard overlap with the default box, and (ii) each of the default box that has a jaccard overlap higher than 0.5 with any ground truth box. Otherwise, we assign a negative label to a non-positive default box if its jaccard overlap is lower than 0.5 for all ground truth boxes. The jaccard overlap calculation is as follows:

(4) $$\left(A,B\right)=\frac{\left|A\cap B\right|}{\left|A\cup B\right|}=\frac{\left|A\cap B\right|}{\left|A\right|+\left|B\right|-\left|A\cap B\right|}\in \left[0,1\right]$$

where |A∩B| denotes the intersection of ground truth box and default box, and |A∪B| indicates their union.

In addition, after matching step, positive samples are much more than negative samples, which leads to a significant imbalance between the positive boxes and negative boxes. In order to make the training converge quickly, hard negative mining strategy is applied to balance the ratio of negative and positive training examples to 3:1 at most.

We employ a multi-task loss L(x,c,l,g) on each training batch to jointly optimize multiple vehicle classification and offsets regression from the proposed deep network as the following function:

(5) $$L\left(x,c,l,g\right)=\frac{1}{N}\left(L_{conf}\left(x,c\right)+\alpha L_{loc}\left(x,l,g\right)\right)$$

Here, g is the ground truth box and l is the corresponding predicted box. x denotes whether the matched box (between default box and the ground truth box) belongs to category p, and c indicates the confidence value if x is 1. If the default box is negative, x is 0. The confidence loss $L_{conf}$ is the softmax loss over four classes (i.e., car, bus, van, and others), and is defined as follows:

(6) $$L_{conf}\left(x,c\right)=-\overset{N}{\underset{i\in Pos}{\sum }}x_{ij}^{p}\log\left({\hat{c}}_{i}^{p}\right)-\underset{i\in Neg}{\sum }\log\left({\hat{c}}_{i}^{0}\right),\;\mathrm{where}\;{\hat{c}}_{i}^{p}=\frac{\exp\left(c_{i}^{p}\right)}{\sum_{p}\exp\left(c_{i}^{p}\right)}$$

where $x_{ij}^{p}$ indicates the i-th default box matches the j-th ground truth box with category p, and ${\hat{c}}_{i}^{p}$ represents the probability that the i-th default box belongs to p. For the regression loss $L_{loc}$, we use the Smooth L1 loss [8] to predict the offsets of default box, and is defined as:

(7) $$L_{loc}\left(x,l,g\right)=\overset{N}{\underset{i\in Pos}{\sum }}\underset{m\in \left(cx,cy,w,h\right)}{\sum }x_{ij}^{k}{\mathrm{smooth}}_{L1}\left(l_{i}^{m}-{\hat{g}}_{j}^{m}\right),\;\mathrm{where}\;{\mathrm{smooth}}_{L1}\left(x\right)=\{\begin{array}{c} 0.5x^{2}\;if\left|x\right|<1\\ \left|x\right|-0.5\;\mathrm{otherwise} \end{array}$$

Especially, ${\hat{g}}_{j}^{m}$ is a four-point vector with m∈(cx,cy,w,h), and we apply the following parameterizations of the four coordinates:

(8) $${\hat{g}}_{j}^{cx}=\left(g_{j}^{cx}-d_{i}^{cx}\right)/d_{i}^{w}\;{\hat{g}}_{j}^{cy}=\left(g_{j}^{cy}-d_{i}^{cy}\right)/d_{i}^{h}\;{\hat{g}}_{j}^{w}=\log\left(\frac{g_{j}^{w}}{d_{i}^{w}}\right)\;{\hat{g}}_{j}^{h}=\log\left(\frac{g_{j}^{h}}{d_{i}^{h}}\right)$$

where (cx, cy) is the bounding box center, and w, h are its width and height, respectively. In addition, $\left({\hat{g}}_{j}^{cx},\;{\hat{g}}_{j}^{cy},\;{\hat{g}}_{j}^{w},\;{\hat{g}}_{j}^{h}\right)$ represents the coordinates of the j-th ground truth box, $\left(d_{i}^{w},\;d_{i}^{h}\right)$ is the width and height of the i-th default box, and $l_{i}^{m}$ indicates the offsets of the predicted box relative to the default box.

Furthermore, in Equation (5), the term is normalized by N and weighted by a balancing parameter α. By default, the weight term α is set to 1 by cross validation, and N denotes the number of positive default boxes.

## 4. Experiments and Results

In this section, we evaluate our proposed network DP-SSD for vehicle localization and classification on two public vehicle datasets: the UA-DETRAC dataset [50,51] and the KITTI dataset [52]. Experiments are implemented based on the deep learning framework Caffe [53] and run on Ubuntu 14.04 LTS with an Intel Core i7-7700K CPU @ 4.2 GHz, 32 GB of DDR4 memory and an NVIDIA GeForce GTX 1080Ti GPU.

### 4.1. Dataset and Experimental Configuration

#### 4.1.1. Training Dataset and Data Augmentation

We evaluate our method on two public vehicle datasets. First, we adopt a quarter high definition (960 × 540) UA-DETRAC Benchmark Dataset [50,51] with 40 videos for more than 50,000 real-world vehicle images as the training samples, which are annotated with tight bounding-boxes and multiple vehicle types such as car, bus, van and others. The dataset is captured with an EOS 550D camera (Canon, Beijing and Tianjin, China) at 24 different locations. In detail, the real-world part of the UA-DETRAC dataset is captured under three occlusion status as fully visible, partially occluded by other vehicles and partially occluded by background, and different degrees of truncation as vehicle parts outside the frame. To achieve an even training distribution, the multi-scale vehicles including small (0–50 pixels), medium (50–150 pixels) and large (more than 150 pixels) are used to train our model. Besides, since weather condition is another important factor in vehicle detection, we intentionally employ the dataset with four weather conditions as cloudy, night, sunny and rainy to train our final model.

**Figure 4:**
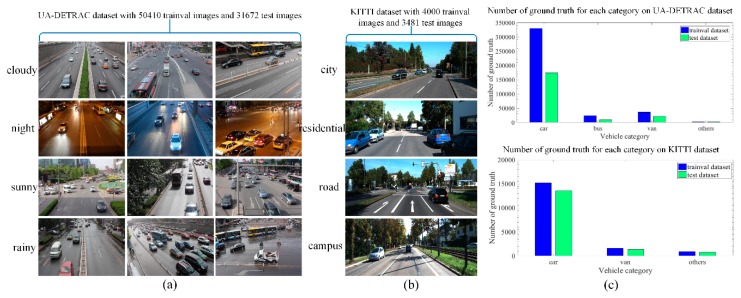
Different datasets for vehicle detection. (**a**) UA-DETRAC dataset: We classify the vehicles into four categories (i.e., car, bus, van, and others) with multiple scales, occlusion ratios and truncation ratios in each weather condition; (**b**) KITTI dataset: We classify the vehicles into three categories (i.e., car, van, and others) with different degrees of occlusion and truncation in multiple scenes; (**c**) Number of ground truth for each category on UA-DETRAC dataset and KITTI dataset, respectively.

To make our model more convincing, we also use a representative (1224 × 370) KITTI Benchmark Dataset [52] with 4000 vehicle images for training. The KITTI dataset utilized here is captured in the mid-size city of Karlsruhe (Germany) under four scenarios: city, residential, road, and campus. Up to 15 vehicles are visible per image, and each bounding box is marked as either visible, semi-occluded, fully occluded or truncated. The examples of the two datasets are illustrated in Figure 4a,b.

For data augmentation, we first randomly sample each of the training data by the options as Reference [12] for making our final model more robust to various vehicle sizes and shapes. In addition, image adjusting brightness, contrast, hue and saturation with probability of 0.5 are introduced to enable that vehicles with blurry edges can be detected as precisely as possible. Then, a strategy of expanding the image ratio is utilized to enhance the classification accuracy of vehicles. Moreover, in order to fully learn the information of vehicles, each training data is horizontally flipped with probability of 0.5.

#### 4.1.2. Implementation Details

The entire training structure of our proposed network is illustrated in Figure 2. We re-scale the images such that their width and height are 300 × 300 pixels. To make a better performance of prediction, we concatenate feature maps of the lower layers through deconvolution to regress vehicle bounding boxes, and concatenate feature maps of the upper layers through pooling to classify vehicle categories. During training, we definitely discover that the enhanced feature extractors can indeed benefit training the network to detect small vehicles. Unfortunately, we remove the BatchNorm layer which ensuring that the feature maps in each concatenated layer are in the same size, due to the consumption of a large amount of memory resources. In the next section, we will provide ablation experiments on different feature pyramids.

For default box, we use four or six different shapes in several feature maps of different resolution with aspect ratios of 1:1, 1:2, 1:3, 2:1, and 3:1. In addition, for the aspect ratio of 1:1, we add another larger scale to resulting in six default boxes per concatenated feature map location. To account for smaller vehicle sizes, we reduce the minimal width and height of default boxes by setting min_ratio as 15 and max_ratio as 90. Figure 3 right shows the capability of our method over different aspect ratios and scales. Table 2 and Table 3 show the learned average default box size for each feature map cell in different concatenated feature layers using the extended VGGNet, with the values of parameters min_size and max_size display on the left. We note that the default box can larger than the underlying receptive field, thus predictions can make adjustment to better match the vehicle shape.

In detail, we directly train the detection and classification network for about 60 epochs on the selected real-world data that contains four vehicle types of car, bus, van and others from the UA-DETRAC dataset. Throughout the training process, the network with 300 × 300 input is trained with a batch size of 24, while 512 × 512 input with a batch size of 8. As in Reference [12], the momentum and weight decay in both nets are configured as 0.9 and 0.0005, respectively. Learning rate in 300 × 300 input net is reduced from 10^−3^ to 10^−5^ by 10^−1^, while in 512 × 512 input net is reduced from 10^−4^ to 10^−6^ by 10^−1^. With each learning rate, we trained 40, 10, 10 epochs, respectively. However, for KITTI dataset with three vehicle types of car, van and others, we first train our models with 10^−4^ learning rate for 480 epochs, followed by 10^−5^ learning rate for 120 epochs, and then continue training for another 120 epochs with 10^−6^, in order to avoid a gradient explosion during training. The other parameter settings are the same as UA-DETRAC dataset. Number of ground truth histograms for each category on UA-DETRAC and KITTI dataset are depicted in Figure 4c.

### 4.2. Experiments on UA-DETRAC Dataset

We comprehensively evaluate our proposed method on the UA-DETRAC Train Images. This dataset consists of 60 sequences with 82,082 real-world vehicle images over four vehicle categories of car, bus, van and others, and we divided it into two parts: 40 sequences with 50,410 images for trainval dataset as mentioned before, and 20 sequences with 31,672 images for test dataset. All methods in this section use the same training and testing data. For the ImageNet pre-trained network, we use the truncated VGG-16 [17] that has 16 convolution layers.

#### 4.2.1. The Importance of Feature Concatenation

We evaluate the performance of DP-SSD by mean average precision (mAP) and frames per second (FPS), respectively.

Table 4 (top) shows DP-SSD results when trained and tested using different size input images. Both DP-SSDs use our new default boxes as adjusted in Table 2 or Table 3. For 300 × 300 input and 512 × 512 input, as shown in Table 5, DP-SSD generates about 8732 and 24,656 default boxes by default settings, respectively. The 300 input model achieves an mAP of 75.43% at 50.47 FPS under our proposed DP-SSD framework. For the 512 input model, there is a 2.51% improvement in accuracy with 77.94% mAP compared to the 300 input model, because more information can be obtained in large images. However, due to the increased computational complexity, the speed is reduced to 25.12 FPS but still achieves in real-time.

To investigate the performance of default box with feature concatenation as a proposal method, we conducted several ablation experiments for comparation. In the first part of the ablation studies, we show the behaviors of different networks with the same 300 × 300 input images. To do this, first, we train the traditional SSD300 model only by replacing the training and testing datasets with UA-DETRAC dataset, which achieves a lower mAP of 74.18% and a higher FPS at 58.78 than DP-SSD300 input model. We observe that this is because of weak feature pyramid and relatively simple computations for vehicle detection. We use this result as a baseline for the following comparisons.

Next, we separately investigate the results of single feature concatenation for pooling concatenation and deconvolution concatenation outputs by eliminating either of them in DP-SSD framework. When feature concatenation through deconvolution is removed (thus the representation power of feature pyramid for vehicle localization is the same as SSD), it results in mAP of 73.49% which is 0.69% lower than conventional SSD. In addition, the speed drops to 52.36 FPS.

On the other hand, when the feature concatenation through pooling is removed in both training and testing time (so the feature pyramid for vehicle categorization becomes conventional SSD), the mAP drops to 73.27%, and the speed is degraded to 51.78 FPS. Somewhat surprisingly, the enhanced feature pyramids in these two models have not played a positive role, the reason can be speculated that unilaterally enhancing low-level or high-level feature pyramids in conventional SSD can make a great difference in computational complexity between localization task and categorization task, resulting in inadequate learning of different tasks in a single network.

Then, we also evaluate the roles of simultaneous integration of deconvolution and pooling. For this purpose, we train a DP-SSD model that only enhances the feature pyramid without adjusting the default box scales. It is observed that the mAP improves from 74.18% (conventional SSD) to 74.77% (DP-SDD using feature concatenation only), but still 0.66% lower than 75.43% (our DP-SSD using feature concatenation and new default box scale), indicating that our feature concatenation method can effectively improve the detection result because of the features for both localization and categorization have been properly learned. Moreover, this shows that the adjusted default box scale in Table 2 can also account for the accuracy of our DP-SSD model, because the default boxes in Table 2 have a wider range. In addition, the speed drops to 49.22 FPS, because this model performs more operations than conventional SSD.

For the 512 input models, in the second part of the ablation experiments, we observe that they have the similar variation tendency as the 300 input models. At 27.75 FPS, conventional SSD gets 76.83% mAP, while the two single feature concatenation models (pooling concatenation and deconvolution concatenation) decrease by 0.96% at 26.79 FPS and 1.63% at 25.83 FPS, respectively. For the DP-SDD using feature concatenation only, there is a 0.21% improvement in accuracy with 77.04% mAP compared to traditional SSD.

Looking at the results from Table 4, the 512 input models achieve higher accuracy than the corresponding 300 input models due to more information can be learned in the larger input images. As for speed, although still reach real-time, the 512 input models are significantly reduced, because of the increased computational complexity. It is noteworthy, both DP-SSD300 and DP-SSD512 obtain the best accuracy at real-time, indicating that our feature concatenation method and default box adjustment strategy have played a positive role.

Table 5 shows the experimental results of three types of DP-SSD for different default box settings. For the 300 input models, the first one, which checks 4 or 6 default boxes per feature map location for a total of 8732 boxes, results in mAP of 75.43%. In the model with 4 default boxes for each layer with a 7760 total boxes, the mAP drops to 75.10%, likewise, for the model which makes a 11,640 total boxes, the mAP becomes 73.62%. For the 512 input models, the model contains 24,656 boxes in total achieves the best mAP of 77.94%. In the case of a 21,968 and a 32,952 total boxes, it has 2.34% and 1.4% mAP drops when they use 4 and 6 default boxes, respectively. All these demonstrate that the different number of boxes for each feature layers is better than that of the same number of boxes.

Figure 1b,d shows the qualitative detection results of our proposed DP-SSD300 on UA-DETRAC dataset. As shown in Figure 1b, DP-SSD300 is robust to detect vehicles in real-time for urban traffic surveillance. Furthermore, DP-SSD300 can prevent detecting multiple boxes for one vehicle and improve the detection rate for small vehicles as shown in Figure 1d.

#### 4.2.2. Comparisons with State-of-the-art Detection Methods

Table 6 shows the performance comparison between DP-SSD, Faster R-CNN [9], YOLO [11], YOLOv2 [24], SSD [12], DSSD [25], R-SSD [13], RefineDet [26], SIN [27] and YOLOv3 [29]. In addition to YOLOv3, our DP-SSD512 outperforms all the other methods significantly. Specifically, regardless of the 300 or 512 input model, DP-SSD has better performance than Faster R-CNN in both accuracy and speed. Because DP-SSD eliminates the proposal generation stage and uses a concatenated feature pyramid of different resolutions in a single neural network.

Both the YOLOv2 models (YOLOv2 416 × 416 [24] and YOLOv2 544 × 544 [24]) obviously improve the performance compared to YOLO [11] by 11.3% and 13.44% of mAP respectively due to the elimination of fully connected layers and introduction of anchor boxes, but they are 4.12% and 1.98% lower than DP-SSD512, respectively. In addition, DP-SSD512 obtains an increased mAP of 1.11% compared to state-of-the-art SSD512 detection model [12], and the lower resolution network DP-SSD300 achieves 75.43%, which is worse than SSD512 but is 1.25% higher than SSD300 [12]. Although residual network is utilized by DSSD, there is still a 1.91% mAP degraded in accuracy with 76.03% mAP compared to DP-SSD512. In particular, compared to R-SSD512 and R-SSD300, DP-SSD512 and DP-SSD300 have 0.21% and 0.41% mAP improvement in accuracy, respectively. This suggests that using our proposed feature concatenation method with adjusted default box scales can help improve the vehicle detection results on UA-DETRAC dataset. The other two models, RefineDet (including 320 input and 512 input models) and SIN, produce better results than DP-SSD300, but there is still a small gap with DP-SSD512. Surprisingly, YOLOv3 achieves 88.09% in mAP, which beats all models and is better than DP-SSD512 by 10.15%, the reason is the good residual structure and multi-scale prediction method used in YOLOv3. However, DP-SSD has fewer network parameters than YOLOv3 (138M vs 214M), thus it is easy to train. As for speed, all models except Faster R-CNN [9], DSSD (ResNet-101) [25], and SIN [27] in Table 6 can detect vehicles in real-time, and YOLOv2 [24] outperforms all other methods with a processing speed of 64.65 FPS.

**Figure 5:**
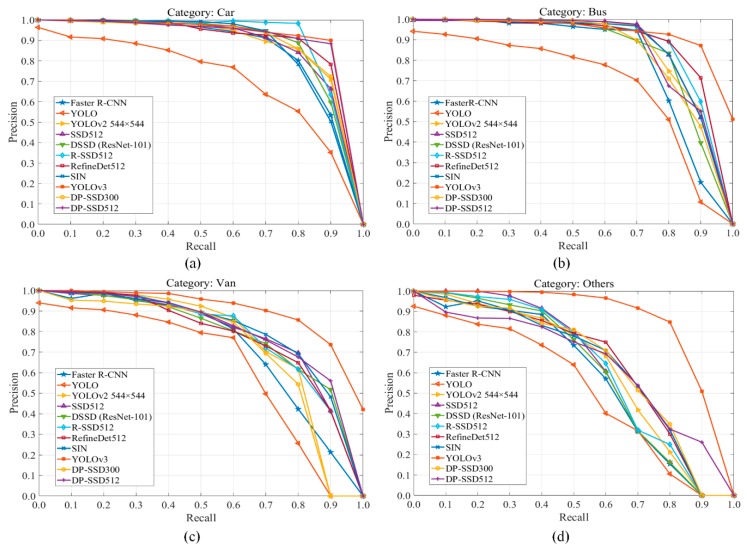
Precision-recall curves between different methods on UA-DETRAC dataset over four categories of car, bus, van, and others. (**a**) Precision-recall curves for the car category; (**b**) Precision-recall curves for the bus category; (**c**) Precision-recall curves for the van category; (**d**) Precision-recall curves for the others category.

**Figure 6:**
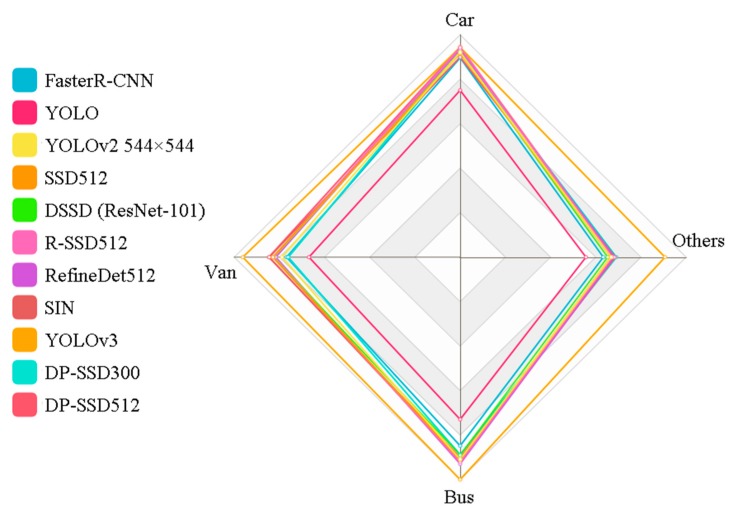
Radar chart of AP values for four vehicle categories of car, bus, van, and others, to show the ability of each model for detecting each type of vehicle.

In Figure 5, we present the precision-recall curves of all the compared models on UA-DETRAC dataset for categories of car, bus, van and others, respectively. We can see, YOLOv3 achieves the best performance with obvious gaps in all four categories by comparing Area Under the Curve (AUC). In addition to YOLOv3, for category car and van, we can further discover that, DP-SSD512 obtains better performance than other methods. Meanwhile, compared with DP-SSD512, DP-SSD300 and RefineDet512 perform slightly better in the others and bus categories, respectively. Specifically, the capacity to detect for each category over these methods is shown in Figure 6. It can be observed that only YOLOv3 has a relatively balanced ability to detect all types of vehicles, while YOLO has relatively poor performance on each type. Apart from this, all the other methods are insufficient for handling the others category, due to the uncertainty of vehicle shapes and the lack of training samples. In our on-going work, we are exploring data augmentation techniques, such as Generative Adversarial Networks, to improve the detection effect of DP-SSD.

### 4.3. Experiments on KITTI Dataset

We further evaluate our DP-SSD300 and DP-SSD512 architectures on the KITTI Images. The dataset we utilized here consists of 7481 images, and we divided it into two parts: 4000 images for the trainval dataset as mentioned before, and 3481 images for the test dataset. 

After training, we test our final model on the KITTI test set with 7518 images, and upload test files to the KITTI website for results at three levels: easy, moderate and hard. We use the same framework as those for UA-DETRAC dataset.

**Figure 7:**
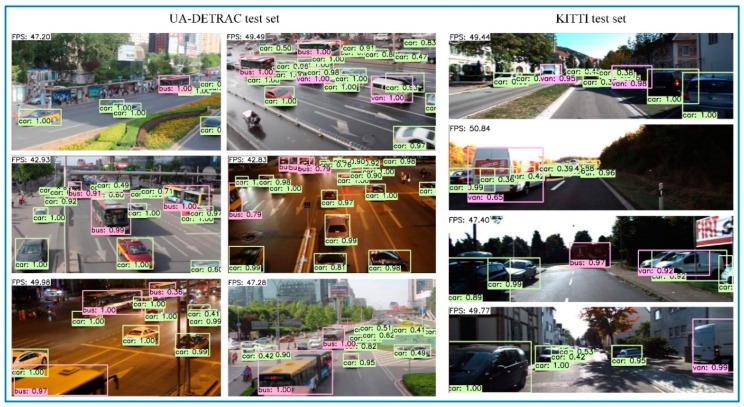
Selected examples of vehicle detection results on UA-DETRAC test set and KITTI test set using our proposed DP-SSD300 framework. Both UA-DETRAC and KITTI test sets are made into video sequence for testing. We display high quality detections with scores higher than 0.3. The detected frame rate is displayed in the upper left corner, and each output box is associated with a category label and a specific score.

Table 7 shows the results of our DP-SSD300 and DP-SSD512 model on KITTI test set. Similar to what we observed on UA-DETRAC dataset, DP-SSD512 achieves 85.32% in mAP on the moderate case, which outperforms R-SSD512 by 0.61%. Likewise, DP-SSD300 can also obtain a relatively high mAP of 83.86%, which is better than R-SSD300. It is clear that both DP-SSD512 and DP-SSD300 perform better than some existing methods in accuracy. However, compared to the best method (e.g., THU CV-AI) published on the KITTI website, there is still a large gap in DP-SSD. Meanwhile, our methods obtain a relatively fast inference speed. In Figure 7, we show some qualitative detection examples on UA-DETRAC and KITTI test sets with the DP-SSD300 model. It can be observed that our proposed method is robust to detect vehicles with different categories simultaneously for urban traffic surveillance.

## 5. Conclusions

In this paper, we have developed DP-SSD, a single deep neural network for vehicle detection in urban traffic surveillance, which concatenates the feature pyramid of conventional SSD [12] and adjusts the scales of default box to detect small vehicles more accurately. The improved detection network predicts bounding-boxes with scores for each vehicle and infers their categories simultaneously. Extensive experiments on the UA-DETRAC and KITTI datasets show that our method outperforms some existing algorithms on accuracy and achieves real-time detection. One of the shortcomings of this paper is that due to the limitations of GPU memory, the batch normalization layer was removed, which is considered to speed up the network training and improve the accuracy of the model. In our on-going work, we are adding multi-label classification task into DP-SSD to simultaneously learn more vehicle attributes such as color, make and model, and ultimately investigate vehicle re-identification tasks in urban traffic surveillance images.

## Figures and Tables

**Table 1:** Network architectures of the feature concatenation through deconvolution and pooling.

	Type	Layer_Name	Bottom	Top
Feature concatenation through deconvolution for localization				
Stage 1	Convolution	conv9_2	conv9_1	conv9_2
Stage 2	Deconvolution	deconv9_2	conv9_2	deconv9_2
	Concat	conv8_2_concat	deconv9_2 & conv8_2	conv8_2_concat
Stage 3	Deconvolution	deconv8_2	conv8_2_concat	deconv8_2
	Concat	conv7_2_concat	deconv8_2 & conv7_2	conv7_2_concat
Stage 4	Deconvolution	deconv7_2	conv7_2_concat	deconv7_2
	Concat	conv6_2_concat	deconv7_2 & conv6_2	conv6_2_concat
Stage 5	Deconvolution	deconv6_2	conv6_2_concat	deconv6_2
	Concat	fc7_concat	deconv6_2 & fc7	fc7_concat
Stage 6	Deconvolution	defc7	fc7_concat	defc7
	Concat	conv4_3_concat	defc7 & conv4_3	conv4_3_concat
Feature concatenation through pooling for categorization				
Stage 1	Convolution	conv4_3	conv4_2	conv4_3
Stage 2	Pooling	pooling4_3	conv4_3	pooling4_3
	Concat	fc7_concat	pooling4_3 & fc7	fc7_concat
Stage 3	Pooling	poolingfc7	fc7_concat	poolingfc7
	Concat	conv6_2_concat	poolingfc7 & conv6_2	conv6_2_concat
Stage 4	Pooling	pooling6_2	conv6_2_concat	pooling6_2
	Concat	conv7_2_concat	pooling6_2 & conv7_2	conv7_2_concat
Stage 5	Pooling	pooling7_2	conv7_2_concat	pooling7_2
	Concat	conv8_2_concat	pooling7_2 & conv8_2	conv8_2_concat
Stage 6	Pooling	pooling8_2	conv8_2_concat	pooling8_2
	Concat	conv9_2_concat	pooling8_2 & conv9_2	conv9_2_concat

**Table 2:** The learned average default box size for each feature map cell in different concatenated feature layers using the extend VGGNet (300 × 300 input).

Layer	Min_size	Max_size	1:1 (Small)	1:1 (Large)	1:2	2:1	1:3	3:1
conv4_3_pb	21	45	21 × 21	31 × 31	15 × 30	30 × 15	-	-
fc7_pb	45	99	45 × 45	67 × 67	32 × 64	64 × 32	26 × 78	78 × 26
conv6_2_pb	99	153	99 × 99	123 × 123	70 × 140	140 × 70	57 × 171	171 × 57
conv7_2_pb	153	207	153 × 153	178 × 178	108 × 216	216 × 108	88 × 265	265 × 88
conv8_2_pb	207	261	207 × 207	232 × 232	146 × 293	293 × 146	-	-
conv9_2_pb	261	315	261 × 261	315 × 315	185 × 369	369 × 185	-	-

**Table 3:** The learned average default box size for each feature map cell in different concatenated feature layers using the extend VGGNet (512 × 512 input).

Layer	Min_size	Max_size	1:1 (Small)	1:1 (Large)	1:2	2:1	1:3	3:1
conv4_3_pb	36	77	36 × 36	53 × 53	25 × 51	51 × 25	-	-
fc7_pb	77	169	77 × 77	114 × 114	54 × 109	109 × 54	44 × 133	133 × 44
conv6_2_pb	169	261	169 × 169	210 × 210	120 × 239	239 × 120	98 × 293	293 × 98
conv7_2_pb	261	353	261 × 261	304 × 304	185 × 369	369 × 185	151 × 452	452 × 151
conv8_2_pb	353	445	353 × 353	396 × 396	250 × 499	499 × 250	-	-
conv9_2_pb	445	537	445 × 445	489 × 489	315 × 629	629 × 315	-	-

**Table 4:** Detection results on UA-DETRAC test set (trained on UA-DETRAC trainval dataset in this paper). The input size is 300 or 512, but using various detectors for training and testing. * denotes using the new default box size in Table 2 or Table 3.

Method	Input	FPS	mAP (%)	Car	Bus	Van	Others
Ours (DP-SSD) *	300	50.47	75.43	85.38	82.12	70.35	63.87
Ours (DP-SSD) *	512	25.12	77.94	86.27	83.39	78.26	63.84
Aablation experiments follow below:							
SSD [12]	300	58.78	74.18	84.46	81.56	71.85	58.86
Ours (SSD pooling) *	300	52.36	73.49	84.03	79.26	71.76	58.91
Ours (SSD deconvolution) *	300	51.78	73.27	85.10	78.86	70.87	58.26
Ours (DP-SSD)	300	49.22	74.77	84.69	80.54	70.27	63.57
SSD [12]	512	27.75	76.83	84.74	84.32	76.64	61.62
Ours (SSD pooling) *	512	26.79	75.87	85.80	81.26	77.23	59.18
Ours (SSD deconvolution) *	512	25.83	75.15	85.42	77.33	76.91	60.94
Ours (DP-SSD)	512	25.26	77.04	84.87	83.73	76.35	63.21

**Table 5:** Detection results of DP-SSD on UA-DETRAC test set using different number of default boxes in multiple feature maps. The training data is UA-DETRAC trainval dataset in this paper. The multiple feature maps are conv4_3_pb, fc7_pb, conv6_2_pb, conv7_2_pb, conv8_2_pb and conv9_2_pb, respectively. The default setting of using different number of default boxes (75.43% or 77.94%) is the same as that in Table 4. All models use the new default box size in Table 2 or Table 3.

Method	Input	Settings	Default Boxes in Multiple Feature Maps	Total Boxes	mAP (%)
Ours (DP-SSD)	300	default	4 6 6 6 4 4	8732	75.43
Ours (DP-SSD)	300	all small	4 4 4 4 4 4	7760	75.10
Ours (DP-SSD)	300	all large	6 6 6 6 6 6	11,640	73.62
Ours (DP-SSD)	512	default	4 6 6 6 4 4	24,656	77.94
Ours (DP-SSD)	512	all small	4 4 4 4 4 4	21,968	75.60
Ours (DP-SSD)	512	all large	6 6 6 6 6 6	32,952	76.54

**Table 6:** Detection results between different methods on UA-DETRAC dataset. In addition to YOLOv3, DP-SSD512 outperforms all other methods on accuracy in real-time, and YOLOv2 achieves the highest speed of 64.65 FPS. * denotes using the new default box size in Table 2 or Table 3.

Method	Input	mAP (%)	FPS
Faster R-CNN [9] (VGG16)	-	72.70	11.23
YOLO [11]	448 × 448	62.52	42.34
YOLOv2 [24]	416 × 416	73.82	64.65
YOLOv2 544 × 544 [24]	544 × 544	75.96	39.14
SSD300 [12]	300 × 300	74.18	58.78
SSD512 [12]	512 × 512	76.83	27.75
DSSD (ResNet-101) [25]	321 × 321	76.03	8.36
R-SSD300 [13]	300 × 300	75.02	43.78
R-SSD512 [13]	512 × 512	77.73	24.19
RefineDet320 [26]	320 × 320	76.97	46.83
RefineDet512 [26]	512 × 512	77.68	29.45
SIN [27]	-	77.26	10.79
YOLOv3 [29]	416 × 416	88.09	51.26
Ours (DP-SSD300) *	300 × 300	75.43	50.47
Ours (DP-SSD512) *	512 × 512	77.94	25.12

**Table 7:** Detection results between different methods on KITTI test set. All methods are ranked based on the “Moderate”.

Method	mAP (%)			Runtime
	Moderate	Easy	Hard	
THU CV-AI	91.97	91.96	84.57	0.38 s
DP-SSD512 (Ours)	85.32	89.19	74.82	0.1 s
R-SSD512 [13]	84.71	88.96	72.68	0.1 s
DP-SSD300 (Ours)	83.86	87.37	70.78	0.07 s
R-SSD300 [13]	82.37	88.13	71.73	0.08 s
A3DODWTDA (image) [54]	81.54	76.21	66.85	0.8 s
SDP+CRC (ft) [55]	81.33	90.39	70.33	0.6 s
MV3D (LIDAR) [56]	79.76	89.80	78.61	0.24 s
RefineNet [57,58]	79.21	90.16	65.71	0.2 s
Faster R-CNN [9]	79.11	87.90	70.19	2 s
spLBP [59]	77.39	80.16	60.59	1.5 s
Reinspect [60]	76.65	88.36	66.56	2 s
